# Medical College Association

**Published:** 1881-07-20

**Authors:** 


### *MEDICAL COLLEGE ASSOCIATION.*

At the *Medical College Association* in Richmond, on motion of Dr. Bodine, the regular order of business was suspended to enter upon the election of officers. Dr. Gross having stated that he would under no circumstances accept a re-election, Dr. Bodine, of Louisville, was elected president; Dr. Briggs, of Nashville, vice-president, and Dr. Leartes Conner, of Detroit, secretary and treasurer. The hour for the Association having now arrived, the College Association adjourned at 5½ P. M.

At this hour, no quorum being present, the Association was adjourned for the year, subject to the call of the president.

The failure of a quorum at the appointed hour is regarded by many as the death of the College Association. This is not necessarily the case. It may, however, be well regarded as indicative of a loss of confidence in the plan and methods pursued. That good has been accomplished we doubt not, and it is believed that the Colleges which have at heart the elevation of Medical Education will not lapse into indifference or adopt any retrograde measures. And after all, it is scarcely to be expected that the Colleges alone, unsupported by the Journals and by the Profession, can accomplish the reformations so much desired. Years ago we took this ground and urged that the proper basis upon which to work was to secure, through the medical press, the interest and co-operation of preceptors and of the profession generally, that the work might slowly and permanently develop into wholesome and successful results. This we still believe is the plan which should be pursued, and to this end. let a warm and hearty co-operation between the Journalist, the Preceptor and the College Professor, be in every possible manner fostered and encouraged.

